# Suspected acute exacerbation of idiopathic pulmonary fibrosis associated with interferon alpha therapy for hepatitis C: case report

**DOI:** 10.1186/2193-1801-2-101

**Published:** 2013-03-12

**Authors:** Victoria Yee-May Ling, Marianne Mortimore, David J Serisier

**Affiliations:** 1 Department of Respiratory Medicine, Mater Adult Hospital, South Brisbane, Qld 4101 Australia; 2 Department of Gastroenterology, Mater Adult Hospital, South Brisbane, Qld Australia; 3 Mater Health Services, University of Queensland, South Brisbane, Qld Australia; 4 Immunity, Infection and Inflammation Program, Mater Medical Research Institute, Mater Health Services, South Brisbane, Qld Australia

**Keywords:** Idiopathic pulmonary fibrosis / usual interstitial pneumonia, Acute exacerbation of idiopathic pulmonary fibrosis, Interferon alpha, Hepatitis C

## Abstract

Interferon alpha (IFNα) has immune stimulatory actions implicated in its pulmonary toxicities. We describe deterioration of idiopathic pulmonary fibrosis (IPF) associated with IFNα treatment for chronic hepatitis C in a 58 year old woman culminating in a fatal suspected acute exacerbation of IPF (AE-IPF). Caution should be exercised in the use of IFNα in subjects with concomitant IPF given its known immunostimulatory effects and possible role in this suspected AE-IPF.

## Background

Pulmonary toxicity is a recognised complication of interferon alpha (IFNα), thought to be related to its immunostimulatory effects. IFNα has been shown to influence the immune system along multiple pathways including augmentation of proinflammatory and profibrotic cytokines (Midturi et al. 2004). Interstitial pneumonitis is the most commonly documented pulmonary toxicity of IFNα (Midturi et al. 2004), but potential deterioration of existing interstitial lung disease needs to be highlighted. We describe a case of progression of usual interstitial pneumonia (UIP)/idiopathic pulmonary fibrosis (IPF) during IFNα treatment, which culminated in a fatal acute exacerbation of IPF (AE-IPF).

### Case description

**Figure 1:**
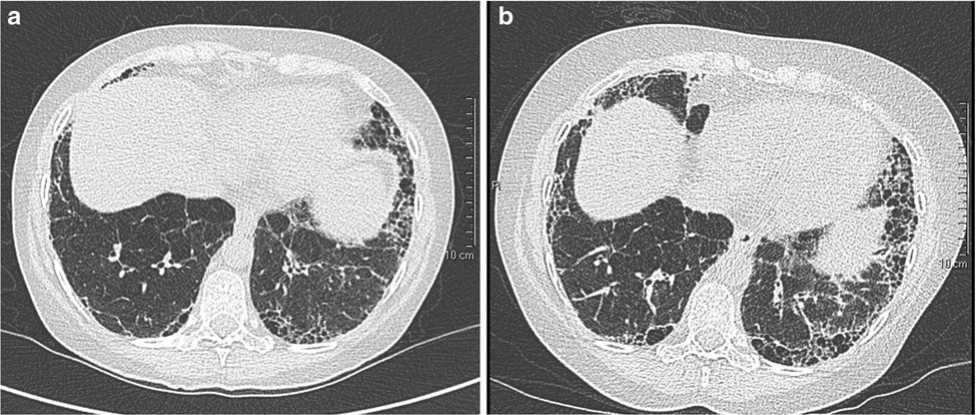
**Serial chest HRCT images in relation to IFNα therapy.** Progression of subpleural interstitial fibrosis and honeycombing from (**a**) 9 months prior to the current presentation, prior to commencement of IFNα to (**b**) 3 months prior, 2 months after commencing IFNα therapy.

A 58 year old woman with probable UIP/IPF and hepatitis C virus (HCV)-related Child-Pugh A liver cirrhosis developed an oesophageal variceal bleed. Hepatitis C was associated with moderate inflammatory activity (Metavir score A3F4), previous hepatic decompensation with ascites and F3 oesophageal varices treated with prophylactic ligation. She had a history of tobacco-related chronic obstructive pulmonary disease (COPD). Eighteen months before admission, high-resolution computed tomography (HRCT) of the chest demonstrated lower zone predominant subpleural interstitial fibrosis with honeycombing consistent with UIP/IPF (see Figure 1a), in addition to centrilobular emphysema.

Five months before admission, she commenced pegylated-interferon alpha-2α and ribavirin. Lung function tests repeated after 1 month of therapy showed 6% reduction in forced vital capacity (FVC) (see Table 1), unassociated with ascites at that time. Pulmonary HRCT after 2 months of therapy showed deterioration of fibrotic changes (see Figure 1b).

**Table 1:** **Respiratory function tests of the patient with times in reference to her admission**

	***9 months earlier***	***4 months earlier***
FEV1 - L (% predicted)	2.26 (88)	2.13 (83)
FVC - L (% predicted)	3.60 (111)	3.38 (105)
FEV1/FVC (% predicted)	0.63 (79)	0.63 (80)
TLC – L (% predicted)	5.38 (106)	5.05 (100)
DLCO/VA - mL/min/mmHg/L (% predicted)	1.62 (35)	1.64 (35)

*FEV1*, forced expiratory volume in one second; *FVC*, forced vital capacity; *TLC*, total lung capacity; *DLCO*, carbon monoxide diffusing capacity; VA, alveolar volume.

**Figure 2:**
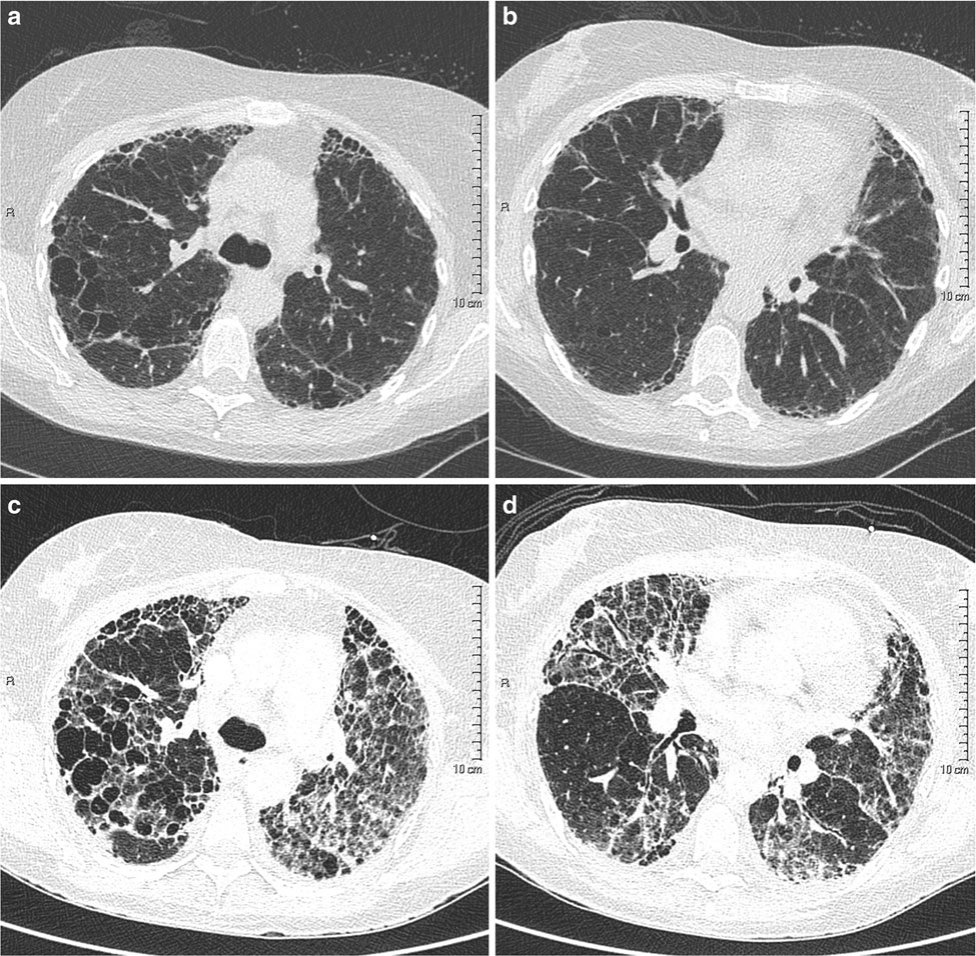
**Serial chest HRCT images in relation to the suspected AE-IPF.** HRCT performed during the exacerbation (**c**) and (**d**) demonstrates diffuse ground glass opacity with marked progression of the underlying fibrosis and honeycombing compared with 3 months prior (**a**) and (**b**).

On admission, she received intravenous octreotide, prophylactic ceftriaxone and endoscopic variceal banding. On day 3, she developed progressive hypoxaemia, requiring high flow nasal prong oxygen to achieve oxyhaemoglobin saturations of 96%. She was febrile to 38.3°C. HRCT of the chest demonstrated diffuse ground-glass change superimposed on markedly deteriorated bilateral pulmonary fibrosis with honeycomb changes (see Figure 2). Intravenous piperacillin/tazobactam, vancomycin, tobramycin and azithromycin were commenced. Sputum specimens cultured *Pseudomonas aeruginosa* and non-multiresistant methicillin-resistant *Staphylococcus aureus* (nmMRSA). On day 6, intravenous hydrocortisone was commenced. On Day 7, bronchoalveolar lavage was performed and specimens again grew nmMRSA, however tested negative for all other pathogens including *Pneumocystis jiroveci* (*carinii*). Transthoracic echocardiogram demonstrated normal left ventricular function. On day 10, there was further deterioration in oxygenation and she required high flow oxygen via mask to achieve oxygen saturations of 90%. Pulse intravenous methylprednisolone was commenced however her respiratory status continued to deteriorate. Escalation of therapy including endotracheal intubation was thought to be futile and she died on day 14.

### Discussion and evaluation

The immunostimulatory properties of IFNα and its recognised risks of pulmonary toxicity including pneumonitis create a strong case for implicating IFNα in potentiating the current deterioration of IPF with a possible contribution to the subsequent suspected AE-IPF. This patient demonstrated objective progression of her probable UIP soon after commencing IFNα, with both physiologic (within 1 month) and radiologic (within 2 months) evidence of deterioration. The 6% decline in FVC was felt to be within the limits of variability, although recent data have shown that FVC decline of >5% predicts a doubling of one year mortality (Zappala et al. 2010).

The acute respiratory decline during the described admission was suspected to be secondary to AE-IPF. Although *P. aeruginosa* and nmMRSA were isolated from respiratory specimens at the time, the patient received appropriate antibiotic therapy promptly at onset (prior to culture of the organisms) and subsequent bronchoscopy (performed because of progressive hypoxia) did not reculture *Pseudomonas.* Furthermore, her clinical course was marked by relentlessly progressive, antibiotic-unresponsive, hypoxic respiratory failure without evidence of sepsis, features much more consistent with AE-IPF than nmMRSA pneumonia. While exclusion of infection forms part of Collard’s proposed criteria for definition of AE-IPF (Collard et al. 2007), infectious agents have been found in up to one-third of subjects with this diagnosis (Borchers et al. 2011) and others have argued that diffuse alveolar damage superimposed upon IPF represents a common endpoint that may be the result of a range of potential triggers, including infection (Corte and Wells 2010).

## Conclusions

Given that IFNα is now part of standard therapy for chronic HCV infection and the rising incidence of UIP/IPF in Western countries (Navaratnam et al. 2011), it is likely that subjects with UIP/IPF will be increasingly exposed to this therapy. The possibility that IFNα may result in significant, potentially fatal deterioration in underlying UIP/IPF necessitates careful consideration prior to embarking upon this therapy in these subjects. Whilst the current case in no way demonstrates causality, we would advise close monitoring and a low threshold for cessation of this therapy in the event of deterioration.

## Abbreviations

AE-IPF: Acute exacerbation of idiopathic pulmonary fibrosis
COPD: Chronic obstructive pulmonary disease
DLCO: Carbon monoxide diffusing capacity
FEV1: Forced expiratory volume in one (1) second
FVC: Forced vital capacity
HCV: Hepatitis C virus
HRCT: High resolution computed tomography
IFNα: Interferon alpha
IPF: Idiopathic pulmonary fibrosis
nmMRSA: Non-multiresistant methicillin resistant *staphylococcus aureus*
TLC: Total lung capacity
UIP: Usual interstitial pneumonia
VA: Alveolar volume
